# Mechanism investigation of highly selective inhibitors toward phosphodiesterase 5 and 6 via the *in vitro* calculation and simulation

**DOI:** 10.3389/fchem.2024.1400886

**Published:** 2024-08-08

**Authors:** Lihang Qu, Kaijian Sun, Zhouyu Jiang, Ting Wang, Linlin Chen, Chunjian Shen, Ruidong Gu

**Affiliations:** ^1^ The 4th People’s Hospital of Shenyang, China Medical University, Shenyang, Liaoning, China; ^2^ School of Pharmacy, Shenyang Pharmaceutical University, Shenyang, Liaoning, China

**Keywords:** phosphodiesterase 5 (PDE5) inhibitors, selectivity, molecular dynamics (MD) simulations, quantum mechanics/molecular mechanics (QM/MM), independent gradient model (IGM) analysis

## Abstract

**Introduction:** In clinical practice, phosphodiesterase 5 (PDE5) inhibitors are commonly used to treat erectile dysfunction and pulmonary arterial hypertension. However, due to the high structural similarity between PDE5 and Phosphodiesterase 6 (PDE6), there is a risk that existing drugs will cause off-target effects on PDE6 resulting in visual disorders such as low visual acuity and color blindness. Previous research on the selectivity of PDE5 inhibitors focused on marketed drugs such as sildenafil and tadalafil.

**Methods:** In this study, a highly selective PDE5 inhibitor, ligand3, was used as the subject, and molecular docking, molecular dynamics simulations, MM-GBSA, alanine scanning, and independent gradient model analysis were employed to investigate the biological mechanism underlying the selectivity of PDE5 inhibitors.

**Results and Discussion:** The present work revealed that the binding mode of ligand3 to the PDE5A and PDE6C targets was distinctly different. Ligand3 exhibited stronger coulombic forces when binding to PDE5A, while showing stronger van der waals forces when binding to PDE6C. Ligand3 binds more deeply at the active site of PDE5A than at PDE6C, allowing its side chains to effectively bind to the critical TYR612, whereas in the case of the shallow binding to PDE6C, ligand3 lacks a similar effect. Mechanism investigations of highly selective inhibitors through computational simulation might provide an insight into potent treatment of drugs.

## 1 Introduction

Phosphodiesterases (PDEs) are isoenzymes distributed in different tissues and cells in the human body, playing a role in regulating the cytoplasmic levels of intracellular second messengers, 3’,5’-cyclic guanosine monophosphate and/or 3’,5’-cyclic adenosine monophosphate. Changes in the quantity of second messengers can lead to various cellular effects in processes such as pathology in the central nervous system, cardiovascular system, inflammation, cell cycle regulation, proliferation, and others (Conti and Beavo, 2007; Omori and Kotera, 2007). PDEs contains 11 families with different encoding genes and substrate specificities (Keravis and Lugnier, 2012). Of these enzymes, PDE5 is mainly distributed in tissues such as vascular smooth muscle of the corpora cavernosa, pulmonary veins, peripheral veins and coronary veins (Hofmann et al., 2000). PDE5 competitively binds with cGMP, interfering with the sexual stimulation mediated by the neurotransmitter nitric oxide (NO), thereby restoring smooth muscle contraction by reducing intracellular Ca2+ concentration. Hence, PDE5 is considered the target enzyme in treating erectile dysfunction (ED) (Corbin et al., 2002; Rotella, 2002).

Currently, PDE5A inhibitors in clinical use include sildenafil, vardenafil, tadalafil, and avanafil. All of these PDE5A inhibitors are used to treat erectile dysfunction, and sildenafil and tadalafil are also used to treat pulmonary arterial hypertension (Corbin et al., 2005; Wespes et al., 2006). Unfortunately, they generally cause dose-dependent side effects, most of which are due to cross-reactivity with other PDEs (Huang and Lie, 2013). PDE6 enzyme is a key effector enzyme in the cascade of light transduction in mammalian rod and cone photoreceptor cells (Marmor and Kessler, 1999; Cote, 2004). It plays a role in visual signal transduction and responds to light through a mechanism of transition from an inactive to an active state, which is regulated by its unique “γ-subunit (Granovsky and Artemyev, 2001; Zhang et al., 2005; Zhang and Artemyev, 2010). The rod PDE6 is a heterotetramer composed of catalytic subunits PDE6A and PDE6B along with two identical inhibitory Pγ subunits, whereas cone PDE6 is a tetramer composed of two identical catalytic subunits PDE6C and two identical inhibitory Pγ subunits (Hamilton and Hurley, 1990; Muradov et al., 2010). Due to the similarity in amino acid sequence and catalytic structural domains between PDE6 and PDE5 (Pissarnitski, 2006), when patients with erectile dysfunction (ED) take PDE5 inhibitors, cross-reactions at the catalytic site of PDE6 can lead to visual disturbances such as functional blindness, cyanopsia, blurred vision, and increased photosensitivity (Foresta et al., 2008; Kerr and Danesh-Meyer, 2009).

**FIGURE 1:**
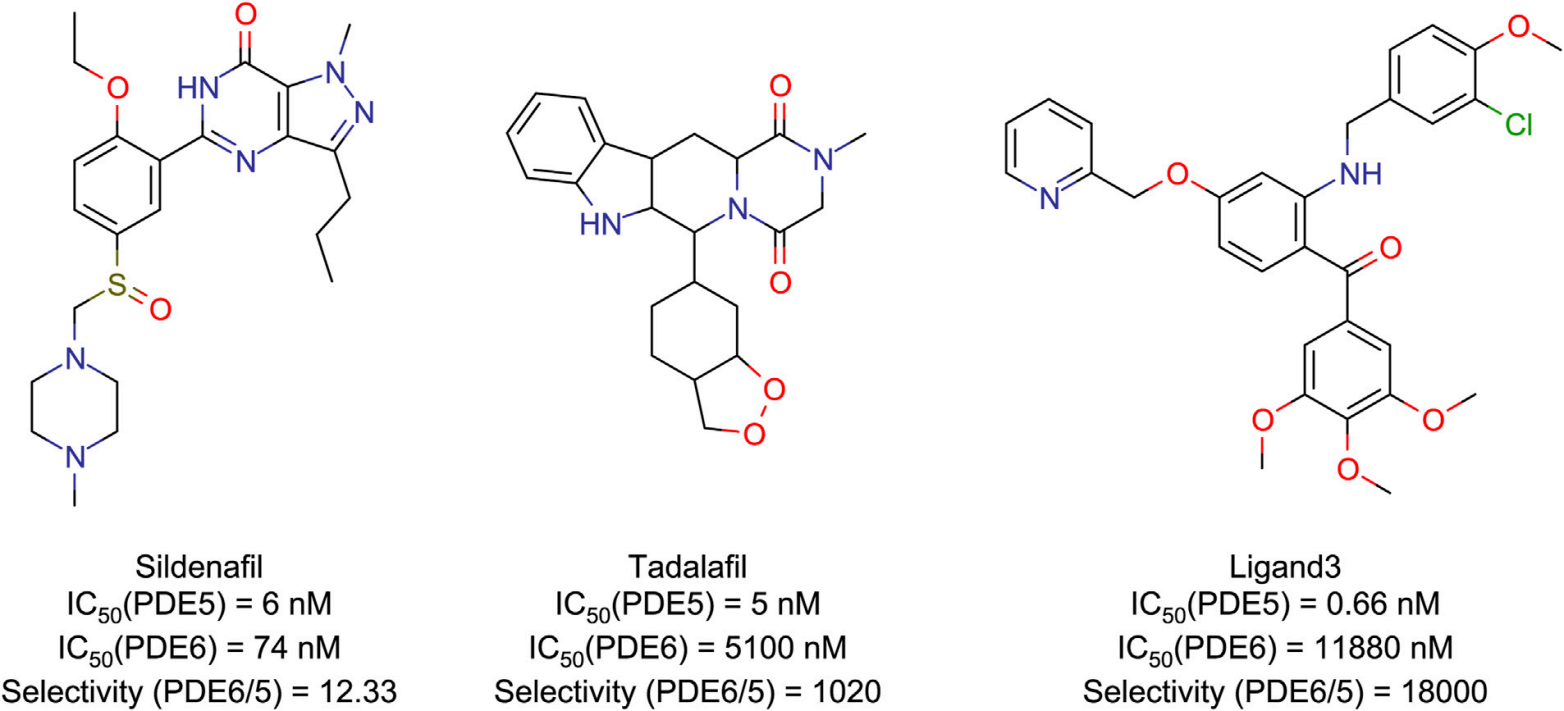
Structures and IC50 (Daugan et al., 2003; Sakamoto et al., 2014) values of PDE5/6 inhibitors.

Although there has been previous research on PDE5 inhibitors (Cahill et al., 2012; Huang et al., 2013; Kayık et al., 2017), past studies have primarily targeted classical inhibitors such as tadalafil and sildenafil, which generally lack strong selectivity. Herein, this study focuses on a novel, highly selective PDE5 inhibitor (Sakamoto et al., 2014) with a selectivity of up to 18,000, along with two classical PDE5 inhibitors (Figure 1). Through the use of molecular docking, molecular dynamics (MD) simulations, quantum mechanics/molecular mechanics (QM/MM), independent gradient model (IGM) analysis, and other simulation methods, there provide a comprehensive understanding of the molecular basis for the high selectivity of PDE5 inhibitors. This research aims to provide theoretical guidance for the design of PDE5 selective inhibitors that can avoid ocular side effects.

## 2 Materials and methods

### 2.1 Model acquisition and evaluation

**FIGURE 2:**
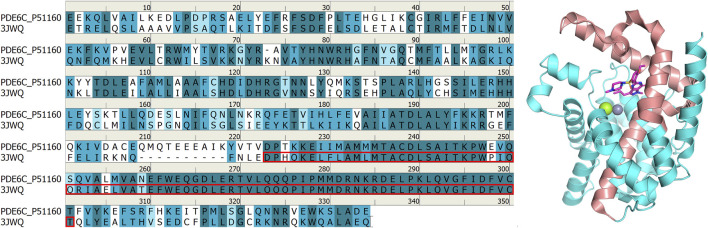
Comparison between 3JWQ sequence and PDB6A sequence (left) and 3JWQ three-dimensional structure (right). The ASP748-THR826 part of 3JWQ at the binding site, which is highly similar to the PDE6C sequence, is marked with red box in the left figure and shown in red in the right figure. In the left figure, perfectly matched residues are shown in dark cyan, residues with strong similarity are shown in cyan, residues with weak similarity are shown in light cyan, and residues without similarity are shown in white. In the right figure, atoms other than carbon are colored according to element type, with magnesium ions shown as green, zinc ions as gray, oxygen atoms as red, nitrogen atoms as blue, and sulfur atoms as yellow.

The protein-ligand complexs crystal file of the catalytic domain of human PDE5A in pdb format (PDB code: 1TBF, 1XOZ) were obtained from RCSB Protein Data Bank (http://www.rcsb.org). The ASP748-THR826 fragment of the chimeric PDE5/PDE6 catalytic domain complexed with sildenafil obtained from the RCSB Protein Data Bank website has a high similarity to the human pde6c sequence (Uniport code: P51160) obtained from Uniport (https://www.uniprot.org/), and this fragment is located at the binding site of sildenafil, which makes it a good reference for constructing three-dimensional models of human PDE6C (Figure 2). Therefore, GLU486-VAL818 fragment in Alphafold (Jumper et al., 2021; Varadi et al., 2022) (https://alphafold.ebi.ac.uk/entry/P51160) structure is taken, and ASP707-VAL777 fragment is replaced by renumber ASP748-VAL818 fragment in 3JWQ structure to obtain the mixed model. The non-conforming PDE6C sequence residues in the model were mutated by Maestro 13.6 in Schrödinger Suite 2023-2 to make the sequence of the hybrid model match the sequence of human PDE6C.

We established the homologous modeling structure of human PDE6C catalytic domain on the Swiss-Model (https://swissmodel.expasy.org/) website using the structure with pdb code 7JSN as the template. All parameters use the site default settings.

The hybrid model and the model obtained from the Swiss-Model website were evaluated with ERRAT (Colovos and Yeates, 1993) and PROCHECK (Morris et al., 1992; Laskowski et al., 1993; Laskowski et al., 1996; Laskowski et al., 2012) separately on the SAVES(https://saves.mbi.ucla.edu/) website.

The models of sildenafil and tadalafil were downloaded from the PubChem website, while the ligand3 model was drawn using Marvin Sketch 24.1.0 (https://www.chemaxon.com) and converted to a 3D structure using Maestro. All sequence Alignment work in this article was performed by the SIM Alignment Tool (https://web.expasy.org/sim/) using the BLOSUM62 comparison matrix with the default values for the web site and demonstrated using the Discovery Studio Visualizer 2019.

### 2.2 Protein and ligand preparation

Protein Preparation Wizard in Schrödinger suite 2023-2 (Schrödinger, LLC, New York, NY, 2023) is used for the preparation of the protein. The hydrogen atoms were added and optimized, atomic clashes were eliminated, formal charges were added to the hetero groups, and then the generated structures were optimized at neutral pH, the pH tolerance for generated structures was set to 2.0. Next, the protonated state of some amino acids is automatically set by Protein Preparation Wizard in a virtual environment with a set pH equal to 7, and further calibrated manually. Hydrogens that had previously been added in random directions were directed in the right direction, depending on the possible formation of hydrogen bonds, to make sure that they were as consistent as possible with the real environment. Finally, the complex structure parameterized with OPLS4 (Harder et al., 2016; Roos et al., 2019) force field is minimized. The ligprep module is used to optimize the structure of small molecules, and the Jaguar module is used to calculate advanced restrained electrostatic potential charges (RESP2) (Schauperl et al., 2020) (δ = 0.6) charges.

### 2.3 Molecular docking

In this study, the Glide program was used to dock three molecules with RESP2 charges onto the structures of PDE5A and PDE6C. The box dimensions were all 30 Å, with the box center taken from the center coordinates of the co-crystallized molecules. Docking was performed with extra precision (XP), and the root mean square deviation (RMSD) were calculated to verify the accuracy of the docking method. Docking scores were used to evaluate the binding strength of ligands.

### 2.4 Molecular dynamics simulations

The complexes formed by the top–scoring compounds in molecular docking were subjected to molecular dynamics simulations until the RMSD fluctuations of the system reached stability. The molecular dynamics simulations were performed using Desmond in the Schrödinger Suite 2023-2, with all complexes parameterized using the OPLS4 force field and placed in periodic rectangular boxes filled with SPC (Berendsen et al., 1981) water. An appropriate amount of Na+ and Cl-ions were added to the boxes to maintain a total charge of 0 and an ion concentration of 0.15 M. The trajectory was recorded at intervals of 100 ps and energy every 1.2 ps. The integration time step was set to 2 fs, and the SHAKE algorithm was used to apply constrain on bonds having hydrogen atoms. The NPT ensemble was used with a temperature of 300 K constrained by the Nosé–Hoover chains (Martyna et al., 1992) thermostat method and pressure of 1.01325 bar constrained by the Martyna-Tobias-Klein (Martyna et al., 1994) barostat method. The cutoff radius of the coulomb interaction is 9 Å. Before the formal dynamics commenced, each system underwent 100 ps of low-temperature Brownian motion MD simulation at 10 K temperature under the NVT ensemble, followed by 12 ps of equilibration under the NPT ensemble with heavy atom constraints, and finally 24 ps of equilibration under the unconstrained NPT ensemble.

### 2.5 Binding free energy calculation

In all the trajectories obtained from MD simulations, 100 frames were extracted respectively and the binding free energy (∆G) is calculated by the Equations below (Equation 1) according to the Molecular mechanics/generalized borne surface area (MM-GBSA) (Knight et al., 2014) method. The MM-GBSA calculation utilized the OPLS4 force field and the implicit solvent model VSGB (Li et al., 2011). The charges of all ligands were not recalculated, and the sampling method employed was Real-Space Minimization. The MM-GBSA method not only calculates the binding free energy to evaluate the binding strength between ligands and proteins, but also quantitatively analyzes the contributions of various energy terms to the binding through energy decomposition

$$∆G_{bind}=∆G_{complex}-\left(∆G_{receptor}+∆G_{ligand}\right)$$ (1)

### 2.6 Alanine scanning mutagenesis analysis

By employing the alanine scanning mutagenesis technique, the difference in binding free energy (ΔΔG) between the receptor and ligand before and after mutating individual residues to alanine within a 5 Å range around the ligand was calculated. This allows for the assessment of the energy contribution of each residue to the binding process. Subtracting the ΔG after mutation from the ΔG before mutation, a positive ΔΔG value indicates that the residue prior to mutation favored the binding of the ligand to the receptor. Residues with significant ΔΔG values are considered key residues at the binding interface.

### 2.7 Hirshfeld surface and IGM

QM/MM is a hybrid computational method that combines the accuracy of QM calculations with the efficiency of MM calculations. In this study, QM calculations were performed on ligands and key residues located in the active site, while MM calculations were applied to other regions to further optimize the conformation of the complex. The QM/MM calculations were performed using the qsite in Schrödinger suite 2023-2. The def2-SVP (Weigend and Ahlrichs, 2005) method and wB97M-V (Mardirossian and Head-Gordon, 2016) functional were used in the QM calculation. The OPLS2005 force field was used for the MM calculation, and the Residue based cutoff distance was set to 12 Å, taking energy and gradient as the convergence criterion.

The hirshfeld surface and Independent Gradient Model (IGM) (Lefebvre et al., 2017; Lu and Chen, 2022) of the computationally optimized structures were analyzed using Multiwfn (Lu and Chen, 2022) to identify weak interactions between the ligand and the receptor, and the Intrinsic Bond Strength Index for Weak Interaction (IBSIW) (Klein et al., 2020) of the residues in the active site was calculated to quantify weak interactions.

The Hirshfeld surface defines the contact surface of a molecule with other molecules, which corresponds to the isosurface of the molecular Hirshfeld weight function value of 0.5. The Hirshfeld weight function can describe the weight of the atom in the three-dimensional space. By mapping the electron density of the atom to the Hirshfeld surface, the region with higher electron density can be identified by the color of the Hirshfeld surface, which corresponds to the stronger interaction.

The difference between IGM and conventional electronic density gradient calculation methods lies in their treatment of electronic density gradient direction. Unlike conventional methods, IGM disregards gradient direction and instead takes the absolute value and sums the electronic density gradients for each atom. Because atomic density gradients do not cancel out in IGM-type density gradient calculations, subtracting the IGM-type density gradient from the conventional electronic density gradient yields δg , which visually highlights regions of overlap in molecular electron clouds, indicating areas of strong interaction between molecules. The IBSIW index is defined as following: (Equation 2).

$$\text{IBSIW}\left(i,j\right)=100\times \frac{\delta g_{i,j}^{pair}}{{\left(d_{i,j}\right)}^{2}}$$ (2)

## 3 Results and discussion

### 3.1 Model acquisition and evaluation

In the SAVES assessment, the Alphafold model, the homology modeling model, and our hybrid model achieved overall quality factor scores of 98.4615, 90.6452 and 94.4615 (Supplementary Data Sheet 2), respectively. The overall quality factor scores for the hybrid model and the Alphafold model were significantly higher than those of the homology modeling model. In the Ramachandran plot, although all residues of the three models fall within the most favored regions and the additional allowed regions, both the Alphafold model (95.8%) and the hybrid model (95.1%) have more residues in the most favored region than homologous models (92.9%) (Supplementary Data Sheet 2). The SAVES evaluation results indicate that both the Alphafold model and the hybrid model are better than homology modeling model. Considering that the small molecule binding region of the hybrid model is taken from the experimental model, we chose the hybrid model as the object for further experiments.

### 3.2 Sequence alignment

**FIGURE 3:**
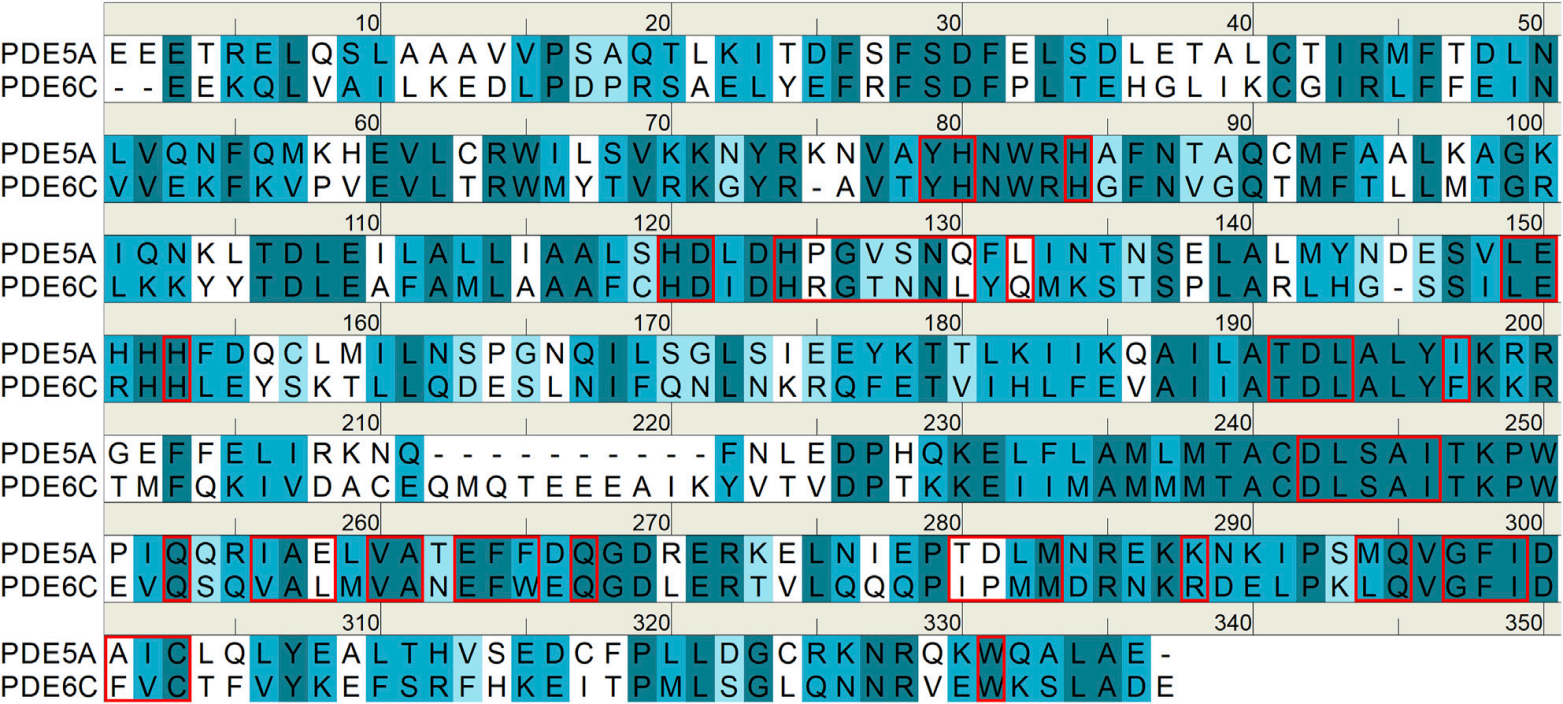
The sequences of PDE5A/6C catalytic domains. Perfectly matched residues are shown in dark cyan, residues with strong similarity are shown in cyan, residues with weak similarity are shown in light cyan, and residues without similarity are shown in white. Residues at active sites are indicated by red boxes.

Sequence alignment has been proven to be an effective method for comparing differences between different target subtypes. The sequence identity between PDE6C and PDE5A is 38.4%, and the sequence similarity is 68.2%. The residues located at the active site in both sequences exhibit high similarity (Figure 3).

### 3.3 Molecular docking

**FIGURE 4:**
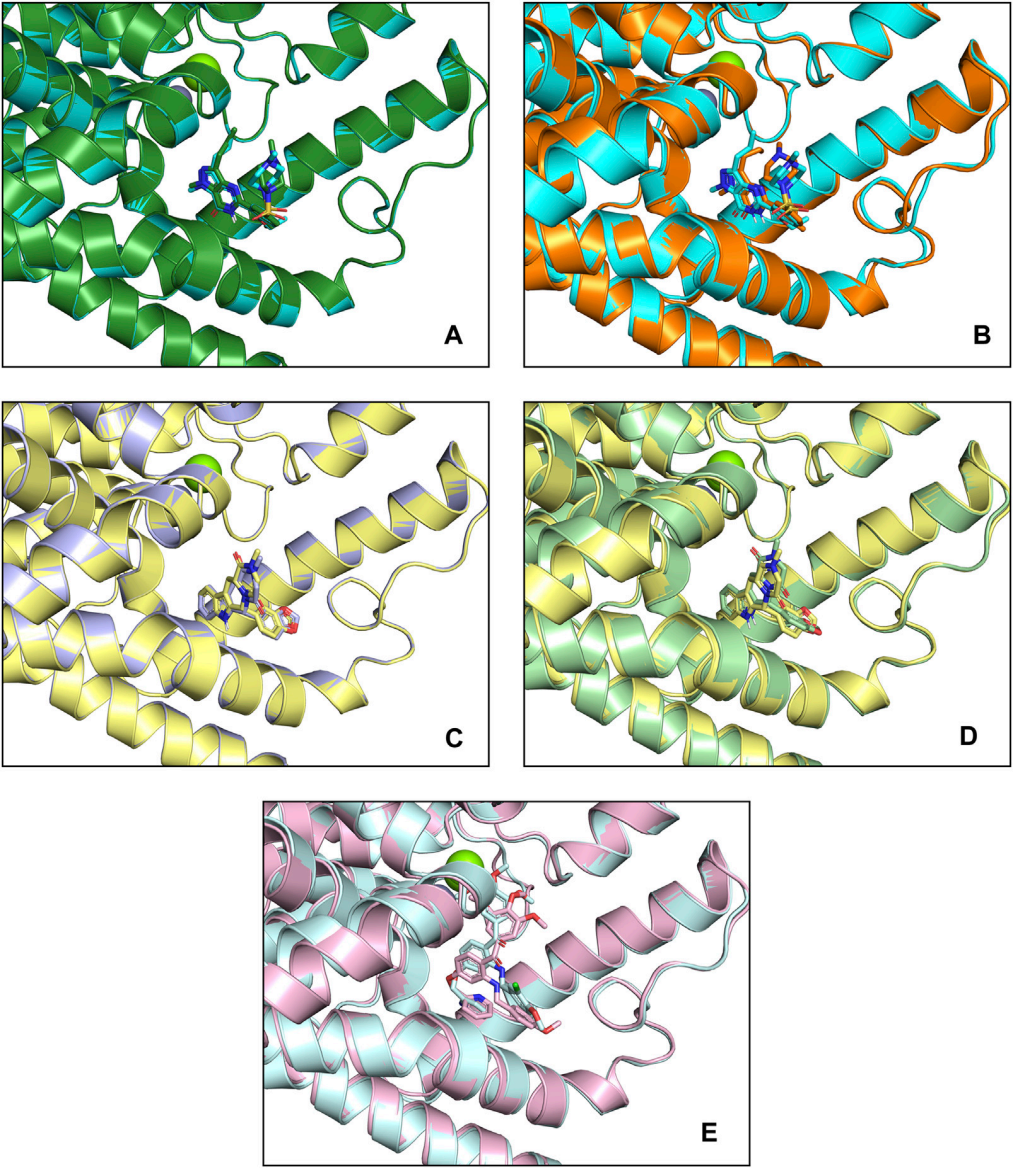
Docking patterns of PDE5A and PDE6C complexes. **(A)** PDE5A (1TBF)-Sildenafil (crystallographic) complexes (deep green) and PDE5A (1TBF)-Sildenafil (docking) complexes (cyan) **(B)** PDE5A (1TBF)-Sildenafil (docking) complexes (cyan) and PDE6A-Sildenafil complexes (orange) **(C)** PDE5A (1XOZ)-Tadalafil (crystallographic) complexes (light blue) and PDE5A (1XOZ)- Tadalafil (docking) complexes (yellow) **(D)** PDE5A (1XOZ)-Tadalafil (docking) complexes (yellow) and PDE6A-Tadalafil complexes (light green) **(E)** PDE5A (1XOZ)-Ligand3 complexes (light pink) and PDE6A-Tadalafil complexes (light cyan).

To tentatively determine the binding modes of small molecules with proteins, three small molecules with RESP2 charges were docked with PDE5A and PDE6C separately. The docking scores and RMSD values of the structures before and after docking are shown in Table 1; Figure 4.

**TABLE 1:** Docking results were utilized to evaluate binding affinity.

Title	Docking score (kcal/mol)	RMSD (Å)	Interaction
PDE5A_Sildenafil	−10.654	0.7552	Hbond: GLN817 π-π stacking: TYR612, PHE786, PHE820
PDE6C_Sildenafil	−10.702	-	Hbond: GLN776 π-π stacking: PHE779
PDE5A_Tadalafil	−9.846	0.4132	Hbond: GLN817 π-π stacking: TYR612
PDE6C_Tadalafil	−8.918	-	Hbond: GLN776 π-π stacking: TYR561, PHE779
PDE5A_Ligand3	−11.097	-	Halogen bond:ALA779 π-π stacking: TYR612, PHE786, PHE820
PDE6C_Ligand3	−9.78	-	Hbond: HIS606 π-π stacking: TYR561, PHE779, Metal Coordination: Mg

Sildenafil’s binding conformation with PDE5A has an RMSD of only 0.7552 Å compared to the crystallographic conformation. Similarly, the RMSD of tadalafil’s binding conformation with PDE5A compared to the crystallographic conformation is 0.4132 Å. This indicates that the docking conformation is essentially consistent with the crystallographic conformation, demonstrating that our docking method can effectively predict the accurate binding mode of the ligand.

**FIGURE 5:**
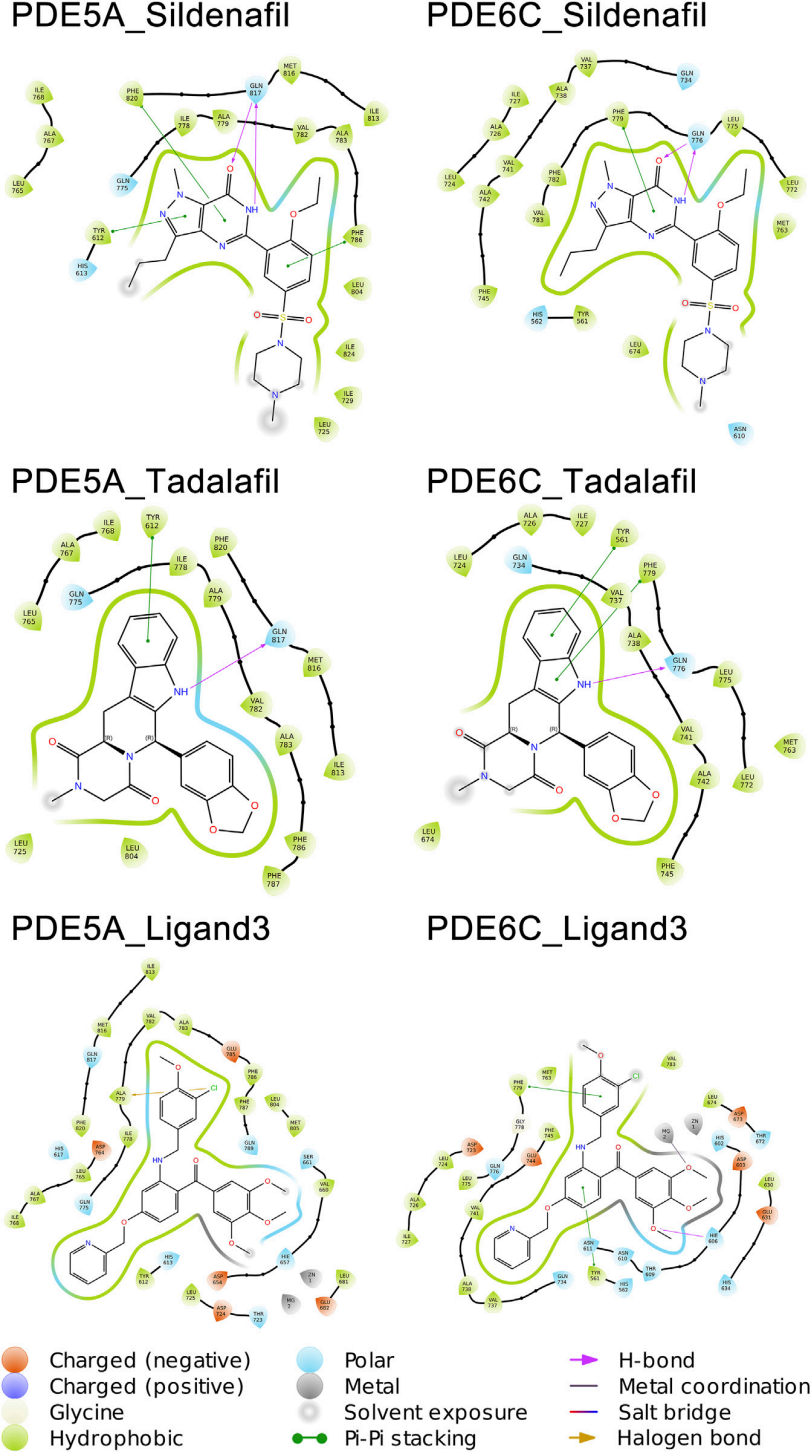
Binding patterns of PDE5A and PDE6C complexes.

Sildenafil achieved similar docking scores and binding modes in both PDE5A and PDE6C. For PDE5A, sildenafil forms hydrogen bonds with GLN817 and π-π stacking with PHE820 and TYR612. Meanwhile, sildenafil forms a hydrogen bond with GLN776 and π-π stacking with PHE779 in PDE6C. Given that sildenafil’s selectivity for both targets is only 12.33, these results are not surprising. Tadalafil and ligand3 have lower docking scores for PDE6C compared to PDE5A, which consistent with the distribution of IC50 values. Similar to sildenafil, tadalafil has almost identical binding modes in both PDE5A and PDE6C. It forms a hydrogen bond with GLN817 in PDE6C, π-π stacking with TYR612, and hydrogen bonds with GLN776 in PDE6C, as well as π-π stacking with PHE779 and TYR561 (Figure 5).

As implied by the significantly different docking scores obtained by ligand3 in binding to PDE5A and PDE6C, there exists a significant difference in its interaction with these two enzymes. The interaction between ligand3 and PDE6C involves hydrogen bonding with HIS606, π-π stacking with PHE779, TYR561 and metal coordination with Mg ions, which are not present in the binding of ligand3 to PDE5A. This highlights the importance of studying ligand3 (Figure 5).

### 3.4 Molecular dynamics simulations

To simulate the dynamic binding of PDE5A selective inhibitors with PDE5A and PDE6C in a realistic environment, MD simulations were performed on six complexes. The simulations were run until the RMSD values of the protein and ligand reached equilibrium (Supplementary Figures S1, S2 in Supplementary Data Sheet 1). For the PDE5A_Sildenafil, PDE6C_Sildenafil, PDE5A_Tadalafil, PDE6C_Tadalafil, PDE5A_Ligand3, PDE6C_Ligand3 complex, MD simulations were performed at 200 ns, 400 ns, 200 ns, 400 ns, 600 ns and 600 ns respectively. Trajectories from the last 100 ns of the simulations were extracted for further analysis.

**FIGURE 6:**
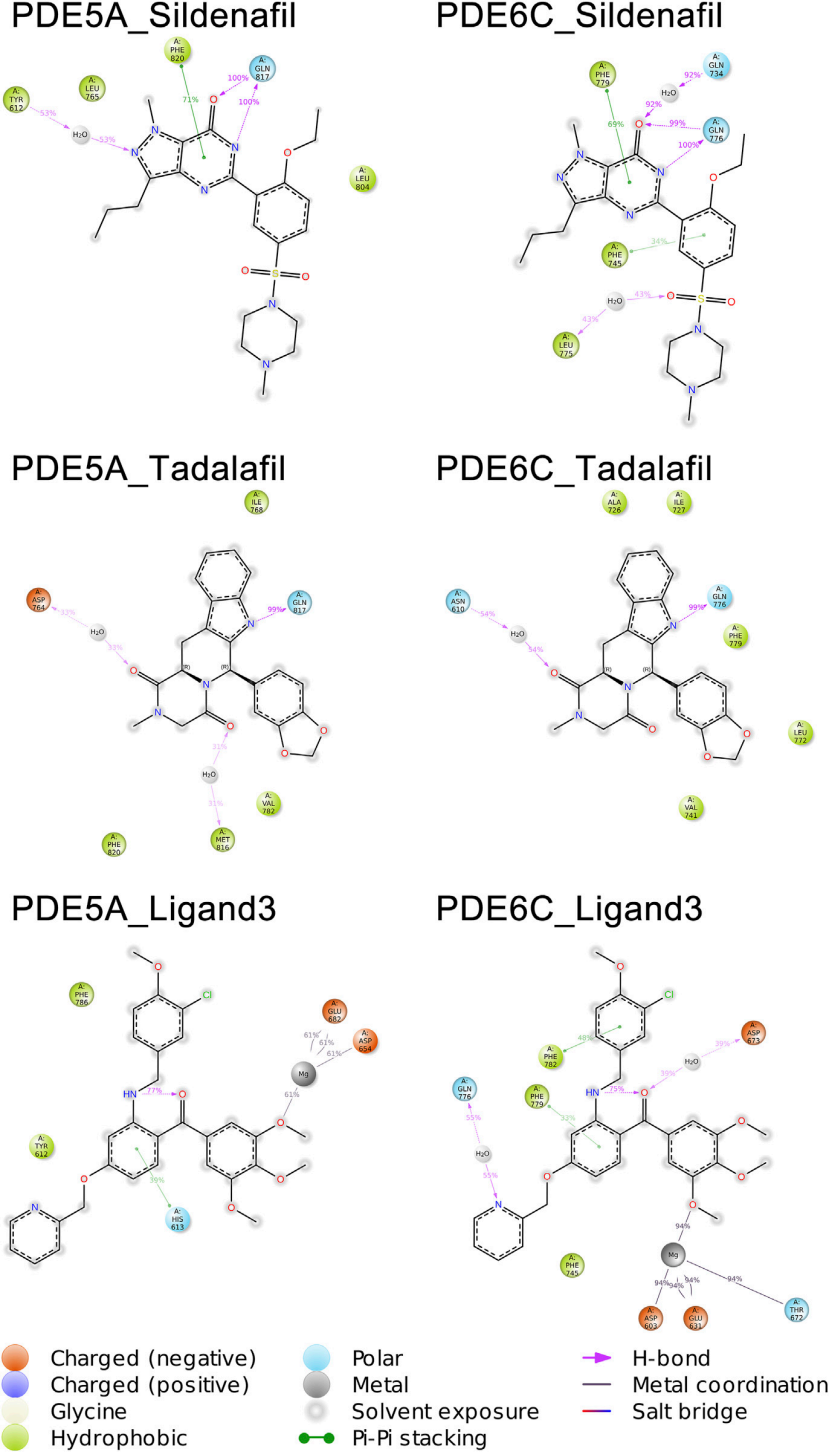
Molecular interactions of PDE5A and PDE6C complexes in MD simulations.

A total of 1,000 complex conformations were extracted from the 100 ns MD simulations trajectory and the mode of interaction between ligand and receptor was analyzed separately. Based on the ratio between the conformation in which a interaction is present and all the extracted conformations, we can infer the percentage of time that this interaction is present in the entire dynamic simulation. (Figure 6) (Supplementary Figures S3, S4 in Supplementary Data Sheet 1).

The mode of action of the PDE5A_Sildenafil complex in molecular dynamics simulations is similar to its mode of action before simulation initiation. In the MD simulation, there is an increase in water bridges with TYR612 and a decrease in π-π stacking with TYR612. The PDE6C_Sildenafil complex in molecular dynamics simulations shows an increase in water bridges with GLN734 and LEU775, as well as π-π stacking with PHE745. It can be observed that the trends in changes for sildenafil in both PDE5A_Sildenafil and PDE6C_Sildenafil complexes are very similar and difficult to distinguish. In the case of the PDE5A_Tadalafil complex, during the MD simulation, tadalafil loses its π-π stacking with TYR612 but gains water bridges with ASP764 and MET816. Similarly, the PDE6C_Tadalafil complex loses its two π-π stacking interactions and gains a water bridge with ASN610 (Figure 5) (Supplementary Figures S3, S4 in Supplementary Data Sheet 1).

During the MD simulation process, the PDE5A_Ligand3 complex gained a new π-π stacking interaction with HIS613 and new metal coordination with a Mg ion. Meanwhile, the PDE6C_Ligand3 complex lost one π-π stacking interaction and one hydrogen bond, but gained water bridges with GLN776 and ASP673, π-π stacking interactions with PHE779 and PHE782, as well as metal coordination with a Mg ion. The mode of action of ligand3 underwent significant changes in both complexes during the MD simulations (Figure 5) (Supplementary Figures S3, S4 in Supplementary Data Sheet 1).

For 100 frames of conformation extracted from MD simulation, the MM-GBSA of ligand and receptor was calculated in VSGB 2.0 (Li et al., 2011) energy model, and the average value of energy was calculated. The results are shown in Table 2. ΔG represents the binding free energy between receptor and ligand, consisting of five energy terms ΔG_Coulomb (Coulomb energy), ΔG_Covalent (Covalent binding energy), ΔG_vdW (Van der Waals energy), ΔG_Lipo (Lipophilic energy), ΔG_Solv (Solvation energy) and three correction phases ΔG_Hbond (Hydrogen-bonding correction), ΔG_Packing (π-π packing correction), ΔG_SelfCont (Self-contact correction) (Table 2).

**TABLE 2:** MM-GBSA algorithm was utlized to calculate average binding free energy.

Title	PDE5A_Sildenafil	PDE6C_Sildenafil	PDE5A_Tadalafil	PDE6C_Tadalafil	PDE5A_Ligand3	PDE6C_Ligand3
ΔG	−78.763	−74.084	−60.581	−66.801	−44.172	−52.769
ΔG_Coulomb	−50.546	−55.448	−9.776	−11.059	−19.492	−12.713
ΔG_Covalent	3.598	4.311	2.256	1.499	4.626	6.989
ΔG_Hbond	−1.698	−1.430	−0.494	−0.507	−0.113	−0.260
ΔG_Lipo	−5.769	−5.410	−13.129	−13.265	−6.240	−7.058
ΔG_Packing	−5.799	−5.576	−3.826	−3.974	−3.834	−5.578
ΔG_SelfCont	0.000	−0.005	0.000	0.014	−0.098	−0.524
ΔG_Solv	41.648	46.860	13.419	14.054	38.273	36.172
ΔG_vdW	−60.198	−57.386	−49.031	−53.563	−57.294	−69.796

^a^All values in the table are given in kcal/mol.

The ΔG of sildenafil with PDE5A is lower than the ΔG of PDE6C, which accords with the IC50 ranking. However, the complex of PDE6C-Tadalafil and PDE6C-Ligand3 has lower ΔG than that of PDE5A, which may be caused by the error of MM-GBSA’s insufficient calculation accuracy. These results suggest the need for further calculations using QM/MM below.

Unlike the similar energy values in complexes of sildenafil and tadalafil, the ΔG_Coulomb of PDE5A_Ligand3 is significantly lower than PDE6C_Ligand3, while the ΔG_vdW of PDE6C_Ligand3 is significantly lower than PDE5A_Ligand3. This to some extent reveals the reason for the selectivity of ligand3, namely, ligand3 binds more to PDE5A through Coulombic forces and more to PDE6C through van der Waals forces.

### 3.5 Alanine scanning mutagenesis analysis

**FIGURE 7:**
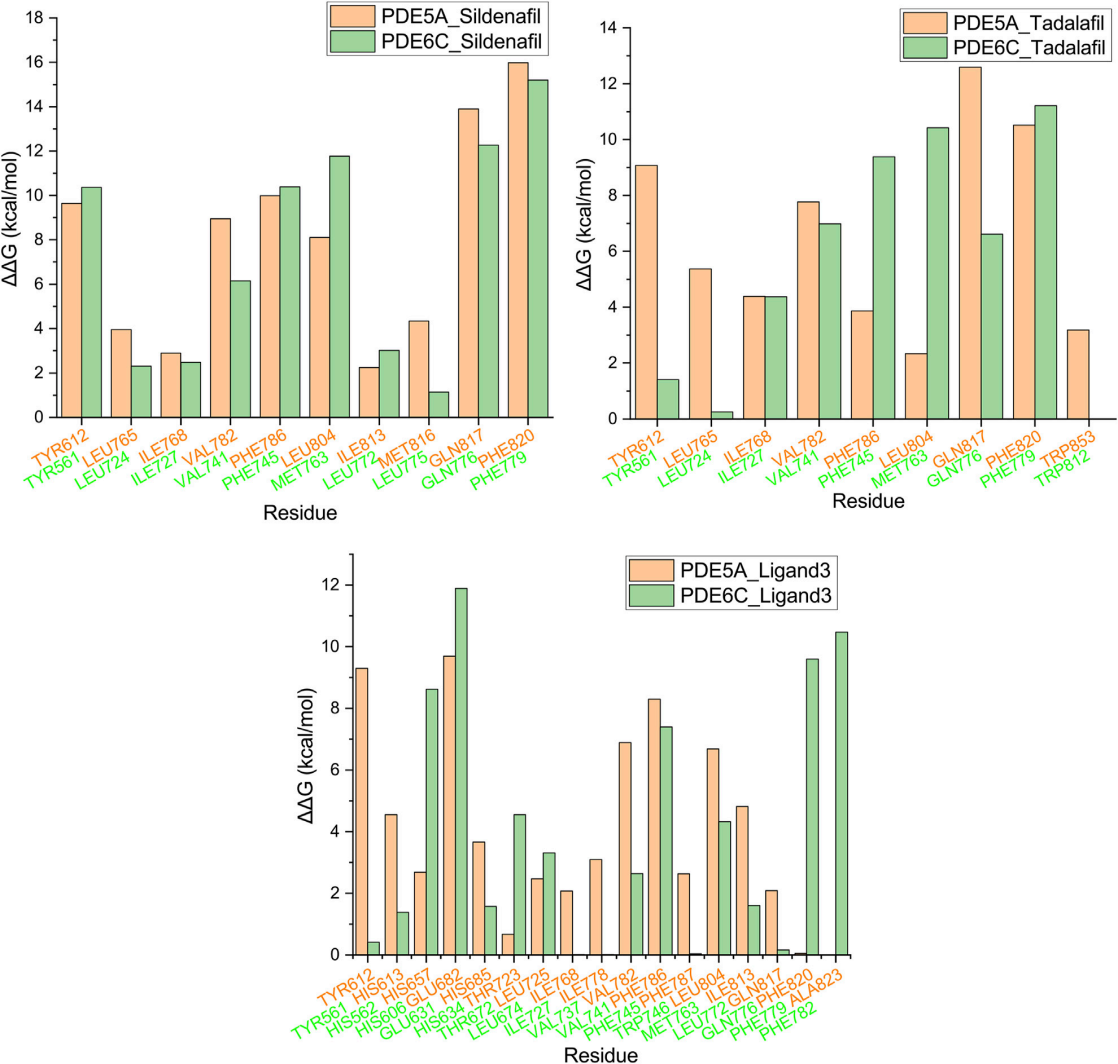
Alanine scanning mutagenesis analysis plot. Orange represents PDE5A, green represents PDE6C, and residues in the same position after superposition are grouped into the same group.

The final conformation of the trajectory, which was sufficiently equilibrated, was used for alanine scanning to identify key amino acids. Residues in the 5 Å range around the ligand are used to calculate the alanine scan. The ΔΔG values of some residues are shown in Figure 7 and Supplementary Table S1. PDE5A_Sildenafil and PDE6C_Sildenafil showed only significant differences greater than or equal to 3 kcal/mol in two residue pairs, LEU804/MET763 and MET816/LEU775, respectively, with the differences almost offsetting each other, which may explain why they have similar ΔG values in the MM-GBSA calculation. PDE5A_Tadalafil had an advantage over PDE6C_Tadalafil at TYR612, LEU765, GLN817, and TRP853, but a disadvantage at PHE786 and LEU804. PDE5A_Ligand3 had an advantage over PDE6C_Ligand3 at TYR612, HIS613, ILE778, VAL782, and ILE813, but a disadvantage at HIS657, THR723, PHE820, and ALA823.

The three molecules, with selectivity values at different magnitudes, exhibited three distinct sets of key amino acids for forming selectivity during alanine scanning in their complexes. This suggests that research on highly selective PDE5A inhibitors cannot be substituted by studies on low-selectivity PDE5A inhibitors, highlighting the necessity to conduct research specifically focusing on molecules with high selectivity.

### 3.6 QM/MM

Due to computational constraints, conventional methods do not involve electronic effects. In order to obtain more accurate binding modes and binding strength data, QM/MM calculations were performed on six complexes, where the key amino acids identified by ligand scanning were set as the QM region while the rest was set as the MM region. Considering the interaction between ligand3 and magnesium ions, the magnesium ions were also included in the QM region in two complexes with ligand3.

**FIGURE 8:**
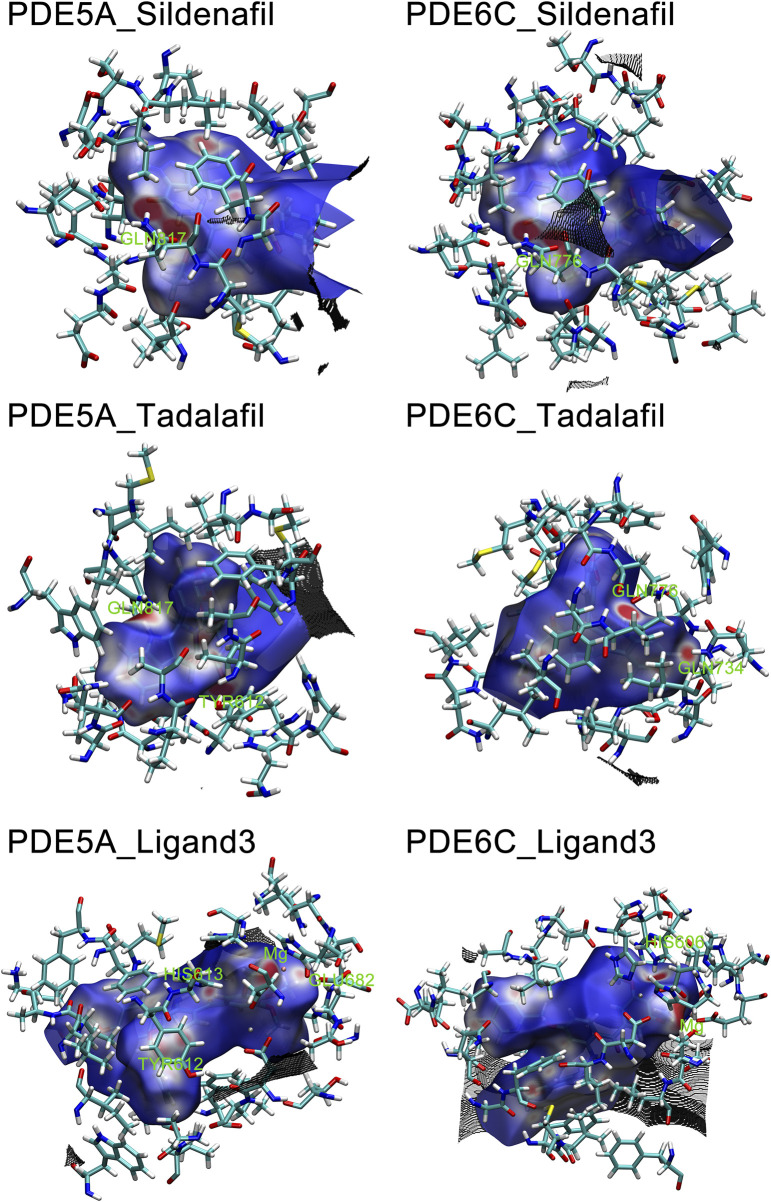
Hirshfeld surface of Ligands in PDE5A and PDE6C complexes. Hirshfeld surfaces were named after F.L Hirshfeld, whose “stockholder partitioning” scheme for defining atoms in molecules suggested to us an extension to defining a molecule in a crystal. Red marks stronger interactions, such as hydrogen bonding, white marks weaker interactions, such as π-π stacking, and blue marks regions close to no interactions.

The hirshfeld surface of the QM/MM optimized complex active site was calculated. Red spots on the hirshfeld surface represent regions with weak interactions, with the size of the red spots indicating the strength of the interactions. In PDE5A_Sildenafil and PDE6C_Sildenafil, the main interacting regions were GLN817/GLN776. In PDE5A_Tadalafil and PDE6C_Tadalafil, GLN817/GLN776 remained the most important regions, but TYR612 in PDE5A_Tadalafil also showed significant interactions, while GLN734 in PDE6C_Tadalafil provided a strong effect. The most prominent interaction in PDE5A_Ligand3 was only with the magnesium ion, whereas in PDE6C_Ligand3, apart from the interaction with the magnesium ion, HIS606 also made a significant contribution to the interactions (Figure 8).

**FIGURE 9:**
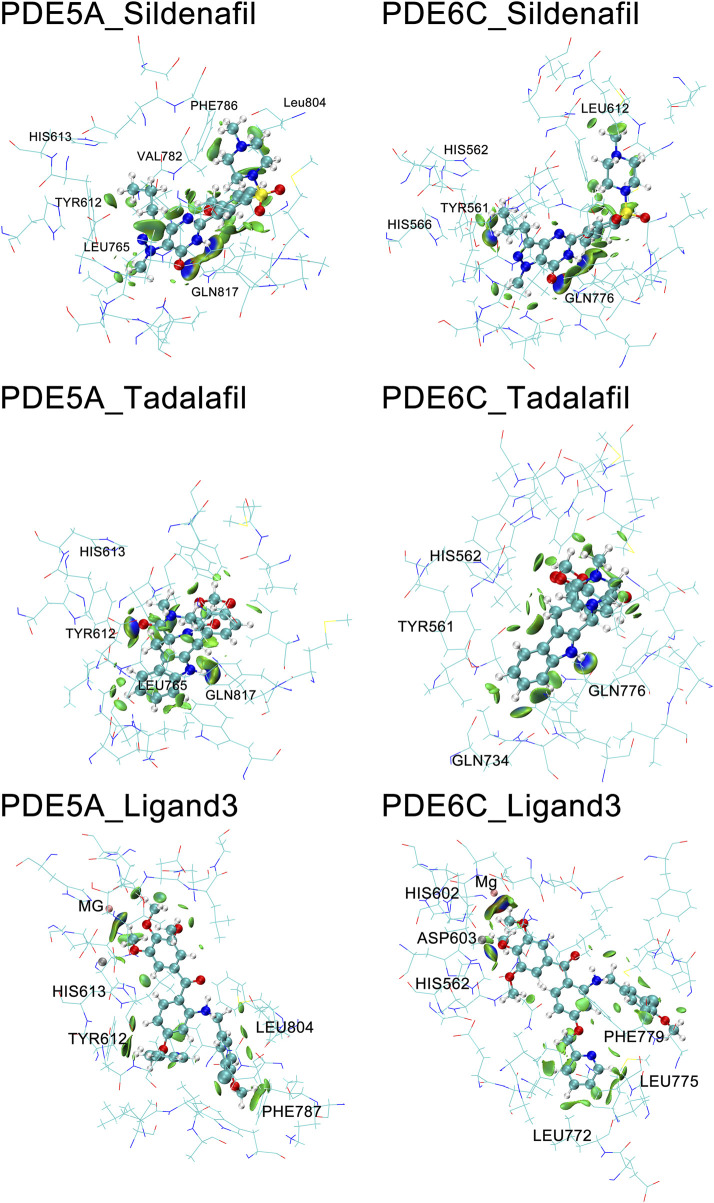
IGM of PDE5A and PDE6C complexes. The surface shows regions with interactions, with blue areas marking stronger interactions and green areas marking weaker interactions.

The IBSIW obtained from IGM analysis can be quantitatively used to calculate the contribution of each residue to the binding of ligands to the receptor. According to Table in supplementary documents (Supplementary Material) and Figure 8, in PDE5A_Sildenafil, TYR612 and HIS613 contributed 43.23% and 24.54% respectively to the binding; in PDE6C_Sildenafil, TYR561, HIS562, and HIS566 contributed 45.95%, 10.43%, and 13.91% respectively to the binding; in PDE5A_Tadalafil, TYR612 and HIS613 contributed 53.48% and 15.06% respectively to the binding; in PDE6C_Tadalafil, TYR561 and HIS562 contributed 50.55% and 16.41% respectively to the binding; in PDE5A_Ligand3, TYR612 and HIS613 contributed 48.58% and 19.27% respectively to the binding; in PDE6C_Ligand3, HIS562 and ASP603 contributed 42.43% and 16.65% respectively to the binding (Figure 9).

In the complexes of sildenafil and tadalafil, the residues with the most prominent contributions are basically the same. However, in the binding of ligand3 to PDE5A and PDE6C, very different residues contribute. This represents the fact that ligand3 has distinctly different binding modes with PDE5A and PDE6C, which undoubtedly leads to differences in binding strength. In PDE5A, TYR612 makes a significant contribution to the binding of ligand3, while the corresponding TYR561 in PDE6C does not play a noticeable role in the binding to ligand3. HIS562 and ASP603 on PDE6C are the most important residues for the binding of ligand3 with PDE6C, whereas their counterparts, HIS613 and ASP654 in PDE5A, only play auxiliary roles. The reason for this difference is that ligand3 can penetrate deeper into the binding site cavity in PDE5A, allowing its side chain to effectively bind with TYR612. In PDE6C, ligand3 cannot interact with TYR561 and instead adopts a mode of binding with HIS562 using 1,2,3-trimethoxybenzene deep at the bottom of the pocket to maintain its binding conformation.

## 4 Conclusion

In this work, we employed strategies such as sequence alignment molecular docking, molecular dynamics simulations, binding free energy calculations, alanine scanning mutagenesis, QM/MM calculations, etc., to investigate the mechanism of selective formation of highly selective PDE5A inhibitors that can avoid ocular side effects. Despite the high similarity of the binding sites of PDE5A and PDE6C, the binding mode of the highly selective PDE5A inhibitor ligand3 shows significant and novel differences in multiple calculations. When ligand3 binds to PDE5A, it exhibits stronger Coulombic force and weaker van der Waals force compared to PDE6C, which may be a key factor in the formation of selectivity. The mechanism of selective formation of highly selective PDE5A inhibitors may differ significantly from that of less selective PDE5A inhibitors. In binding to PDE5A, the pyridine ring region of ligand3 forms a very important role with TYR612. Highly selective PDE5A inhibitors should strengthen side chains that can bind to TYR612 in PDE5A and weaken structures that can bind to HIS562 in PDE6C. In summary, these findings reveal the characteristics and mechanism of selectivity that highly selective PDE5A inhibitors should possess, providing guidance and reference for the future design of selective PDE5A inhibitors that can avoid ocular side effects.

## Data Availability

The raw data supporting the conclusions of this article will be made available by the authors, without undue reservation.

## References

[B1] Berendsen H. J. C. Postma J. P. M. Van Gunsteren W. F. Hermans J. (1981). “Interaction models for water in relation to protein hydration,” in Intermolecular forces: proceedings of the fourteenth Jerusalem symposium on quantum chemistry and biochemistry held in Jerusalem, Israel, april 13–16, 1981. Editor Pullman B. (Dordrecht: Springer Netherlands), 331–342.

[B2] Cahill K. B. Quade J. H. Carleton K. L. Cote R. H. (2012). Identification of amino acid residues responsible for the selectivity of tadalafil binding to two closely related phosphodiesterases, PDE5 and PDE6. J. Biol. Chem. 287, 41406–41416. 10.1074/jbc.m112.389189 23033484 PMC3510839

[B3] Colovos C. Yeates T. O. (1993). Verification of protein structures: patterns of nonbonded atomic interactions. Protein Sci. 2, 1511–1519. 10.1002/pro.5560020916 8401235 PMC2142462

[B4] Conti M. Beavo J. (2007). Biochemistry and physiology of cyclic nucleotide phosphodiesterases: essential components in cyclic nucleotide signaling. Annu. Rev. Biochem. 76, 481–511. 10.1146/annurev.biochem.76.060305.150444 17376027

[B5] Corbin J. D. Beasley A. Blount M. A. Francis S. H. (2005). High lung PDE5: a strong basis for treating pulmonary hypertension with PDE5 inhibitors. Biochem. Biophys. Res. Commun. 334, 930–938. 10.1016/j.bbrc.2005.06.183 16023993

[B6] Corbin J. D. Francis S. H. Webb D. J. (2002). Phosphodiesterase type 5 as a pharmacologic target in erectile dysfunction. Urology 60, 4–11. 10.1016/s0090-4295(02)01686-2 12414329

[B7] Cote R. H. (2004). Characteristics of photoreceptor PDE (PDE6): similarities and differences to PDE5. Int. J. Impot. Res. 16 (Suppl. 1), S28–S33. 10.1038/sj.ijir.3901212 15224133

[B8] Daugan A. Grondin P. Ruault C. Le Monnier De Gouville A.-C. Coste H. Linget J. M. (2003). The Discovery of tadalafil: a novel and highly selective PDE5 inhibitor. 2: 2,3,6,7,12,12a-hexahydropyrazino[1‘,2‘:1,6]pyrido[3,4-b]indole-1,4-dione analogues. J. Med. Chem. 46, 4533–4542. 10.1021/jm0300577 14521415

[B9] Foresta C. Caretta N. Zuccarello D. Poletti A. Biagioli A. Caretti L. (2008). Expression of the PDE5 enzyme on human retinal tissue: new aspects of PDE5 inhibitors ocular side effects. Eye (Lond) 22, 144–149. 10.1038/sj.eye.6702908 17585311

[B10] Granovsky A. E. Artemyev N. O. (2001). A conformational switch in the inhibitory gamma-subunit of PDE6 upon enzyme activation by transducin. Biochemistry 40, 13209–13215. 10.1021/bi011127j 11683629

[B11] Hamilton S. E. Hurley J. B. (1990). A phosphodiesterase inhibitor specific to a subset of bovine retinal cones. J. Biol. Chem. 265, 11259–11264. 10.1016/s0021-9258(19)38585-0 2162841

[B12] Harder E. Damm W. Maple J. Wu C. Reboul M. Xiang J. Y. (2016). OPLS3: a force field providing broad coverage of drug-like small molecules and proteins. J. Chem. Theory Comput. 12, 281–296. 10.1021/acs.jctc.5b00864 26584231

[B13] Hofmann F. Ammendola A. Schlossmann J. (2000). Rising behind NO: cGMP-dependent protein kinases. J. Cell Sci. 113 (Pt 10), 1671–1676. 10.1242/jcs.113.10.1671 10769198

[B14] Huang S. A. Lie J. D. (2013). Phosphodiesterase-5 (PDE5) inhibitors in the management of erectile dysfunction. P t 38, 407–419. 24049429 PMC3776492

[B15] Huang Y. Y. Li Z. Cai Y. H. Feng L. J. Wu Y. Li X. (2013). The molecular basis for the selectivity of tadalafil toward phosphodiesterase 5 and 6: a modeling study. J. Chem. Inf. Model 53, 3044–3053. 10.1021/ci400458z 24180640

[B16] Jumper J. Evans R. Pritzel A. Green T. Figurnov M. Ronneberger O. (2021). Highly accurate protein structure prediction with AlphaFold. Nature 596, 583–589. 10.1038/s41586-021-03819-2 34265844 PMC8371605

[B17] Kayık G. Tüzün N. Durdagi S. (2017). Investigation of PDE5/PDE6 and PDE5/PDE11 selective potent tadalafil-like PDE5 inhibitors using combination of molecular modeling approaches, molecular fingerprint-based virtual screening protocols and structure-based pharmacophore development. J. Enzyme Inhib. Med. Chem. 32, 311–330. 10.1080/14756366.2016.1250756 28150511 PMC6009860

[B18] Keravis T. Lugnier C. (2012). Cyclic nucleotide phosphodiesterase (PDE) isozymes as targets of the intracellular signalling network: benefits of PDE inhibitors in various diseases and perspectives for future therapeutic developments. Br. J. Pharmacol. 165, 1288–1305. 10.1111/j.1476-5381.2011.01729.x 22014080 PMC3372715

[B19] Kerr N. M. Danesh-Meyer H. V. (2009). Phosphodiesterase inhibitors and the eye. Clin. Exp. Ophthalmol. 37, 514–523. 10.1111/j.1442-9071.2009.02070.x 19624350

[B44] Klein J. Khartabil H. Boisson J.-C. Contreras-García J. Piquemal J.-P. Hénon E. (2020). New way for probing bond strength. J. Phys. Chem. A 124, 1850–1860. 32039597 10.1021/acs.jpca.9b09845

[B20] Knight J. L. Krilov G. Borrelli K. W. Williams J. Gunn J. R. Clowes A. (2014). Leveraging data fusion strategies in multireceptor lead optimization MM/GBSA end-point methods. J. Chem. Theory Comput. 10, 3207–3220. 10.1021/ct500189s 26588291

[B21] Laskowski R. Macarthur M. Thornton J. (2012). PROCHECK: validation of protein-structure coordinates. Crystallogr. Biol. Macromol., 684–687. 10.1107/97809553602060000882

[B22] Laskowski R. A. Macarthur M. W. Moss D. S. Thornton J. M. (1993). PROCHECK: a program to check the stereochemical quality of protein structures. J. Appl. Crystallogr. 26, 283–291. 10.1107/s0021889892009944

[B23] Laskowski R. A. Rullmannn J. A. Macarthur M. W. Kaptein R. Thornton J. M. (1996). AQUA and PROCHECK-NMR: programs for checking the quality of protein structures solved by NMR. J. Biomol. NMR 8, 477–486. 10.1007/bf00228148 9008363

[B42] Lefebvre C. Rubez G. Khartabil H. Boisson J. C. Contreras-García J. Hénon E. (2017). Accurately extracting the signature of intermolecular interactions present in the NCI plot of the reduced density gradient versus electron density. Phys. Chem. 19, 17928–17936. 10.1039/c7cp02110k 28664951

[B24] Li J. Abel R. Zhu K. Cao Y. Zhao S. Friesner R. A. (2011). The VSGB 2.0 model: a next generation energy model for high resolution protein structure modeling. Proteins 79, 2794–2812. 10.1002/prot.23106 21905107 PMC3206729

[B43] Lu T. Chen Q. (2022). Independent gradient model based on Hirshfeld partition: a new method for visual study of interactions in chemical systems. J. Comput. Chem. 43, 539–555. 35108407 10.1002/jcc.26812

[B25] Mardirossian N. Head-Gordon M. (2016). ωB97M-V: a combinatorially optimized, range-separated hybrid, meta-GGA density functional with VV10 nonlocal correlation. J. Chem. Phys. 144, 214110. 10.1063/1.4952647 27276948

[B26] Marmor M. F. Kessler R. (1999). Sildenafil (viagra) and ophthalmology. Surv. Ophthalmol. 44, 153–162. 10.1016/s0039-6257(99)00079-x 10541153

[B27] Martyna G. J. Klein M. L. Tuckerman M. (1992). Nosé–Hoover chains: the canonical ensemble via continuous dynamics. J. Chem. Phys. 97, 2635–2643. 10.1063/1.463940

[B28] Martyna G. J. Tobias D. J. Klein M. L. (1994). Constant pressure molecular dynamics algorithms. J. Chem. Phys. 101, 4177–4189. 10.1063/1.467468

[B29] Morris A. L. Macarthur M. W. Hutchinson E. G. Thornton J. M. (1992). Stereochemical quality of protein structure coordinates. Proteins 12, 345–364. 10.1002/prot.340120407 1579569

[B30] Muradov H. Boyd K. K. Artemyev N. O. (2010). Rod phosphodiesterase-6 PDE6A and PDE6B subunits are enzymatically equivalent. J. Biol. Chem. 285, 39828–39834. 10.1074/jbc.m110.170068 20940301 PMC3000964

[B31] Omori K. Kotera J. (2007). Overview of PDEs and their regulation. Circ. Res. 100, 309–327. 10.1161/01.res.0000256354.95791.f1 17307970

[B32] Pissarnitski D. (2006). Phosphodiesterase 5 (PDE 5) inhibitors for the treatment of male erectile disorder: attaining selectivity versus PDE6. Med. Res. Rev. 26, 369–395. 10.1002/med.20053 16388517

[B33] Roos K. Wu C. Damm W. Reboul M. Stevenson J. M. Lu C. (2019). OPLS3e: Extending Force Field Coverage for Drug-Like Small Molecules. J. Chem. Theory Comput. 15, 1863–1874. 10.1021/acs.jctc.8b01026 30768902

[B34] Rotella D. P. (2002). Phosphodiesterase 5 inhibitors: current status and potential applications. Nat. Rev. Drug Discov. 1, 674–682. 10.1038/nrd893 12209148

[B35] Sakamoto T. Koga Y. Hikota M. Matsuki K. Murakami M. Kikkawa K. (2014). Design and synthesis of novel 5-(3,4,5-trimethoxybenzoyl)-4-aminopyrimidine derivatives as potent and selective phosphodiesterase 5 inhibitors: scaffold hopping using a pseudo-ring by intramolecular hydrogen bond formation. Bioorg Med. Chem. Lett. 24, 5175–5180. 10.1016/j.bmcl.2014.09.082 25442307

[B36] Schauperl M. Nerenberg P. S. Jang H. Wang L. P. Bayly C. I. Mobley D. L. (2020). Non-bonded force field model with advanced restrained electrostatic potential charges (RESP2). Commun. Chem. 3, 44. 10.1038/s42004-020-0291-4 34136662 PMC8204736

[B37] Varadi M. Anyango S. Deshpande M. Nair S. Natassia C. Yordanova G. (2022). AlphaFold Protein Structure Database: massively expanding the structural coverage of protein-sequence space with high-accuracy models. Nucleic Acids Res. 50, D439–d444. 10.1093/nar/gkab1061 34791371 PMC8728224

[B38] Weigend F. Ahlrichs R. (2005). Balanced basis sets of split valence, triple zeta valence and quadruple zeta valence quality for H to Rn: design and assessment of accuracy. Phys. Chem. Chem. Phys. 7, 3297–3305. 10.1039/b508541a 16240044

[B39] Wespes E. Amar E. Hatzichristou D. Hatzimouratidis K. Montorsi F. Pryor J. (2006). EAU Guidelines on erectile dysfunction: an update. Eur. Urol. 49, 806–815. 10.1016/j.eururo.2006.01.028 16530932

[B40] Zhang X. Feng Q. Cote R. H. (2005). Efficacy and selectivity of phosphodiesterase-targeted drugs in inhibiting photoreceptor phosphodiesterase (PDE6) in retinal photoreceptors. Invest. Ophthalmol. Vis. Sci. 46, 3060–3066. 10.1167/iovs.05-0257 16123402 PMC1343468

[B41] Zhang Z. Artemyev N. O. (2010). Determinants for phosphodiesterase 6 inhibition by its gamma-subunit. Biochemistry 49, 3862–3867. 10.1021/bi100354a 20397626 PMC2864356

