# Supplementary material for: Mechanism investigation of highly selective inhibitors toward phosphodiesterase 5 and 6 via the in vitro calculation and simulation
Source: Front Chem. 2024 Aug 8;12:1400886. doi: 10.3389/fchem.2024.1400886 (PMC11338870; doi:10.3389/fchem.2024.1400886)
Supplement: Supplementary file 2 [file DataSheet1.PDF]

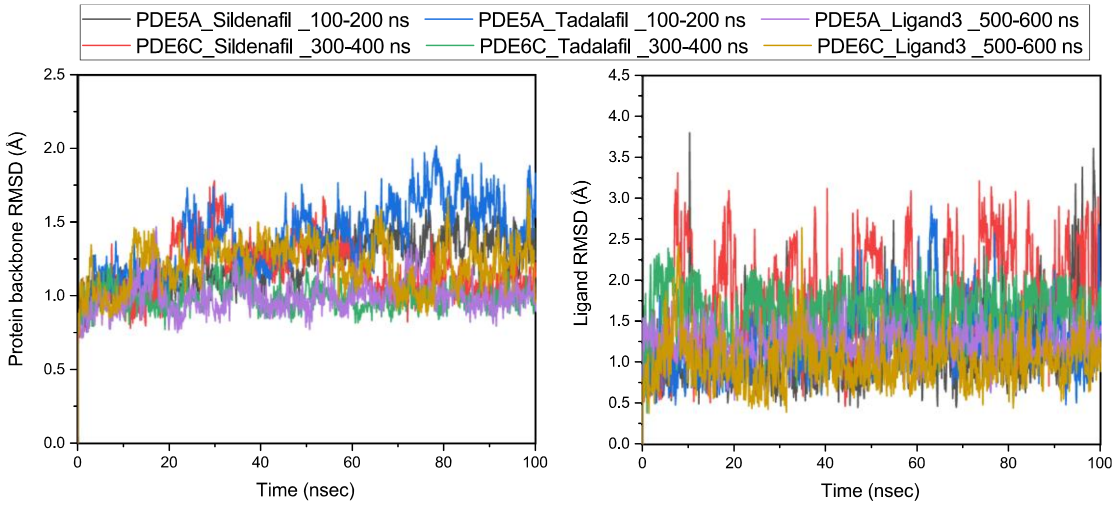

Figure S1 RMSD values of PDE5A and PDE6C complexes throughout the last 100 ns MD simulations

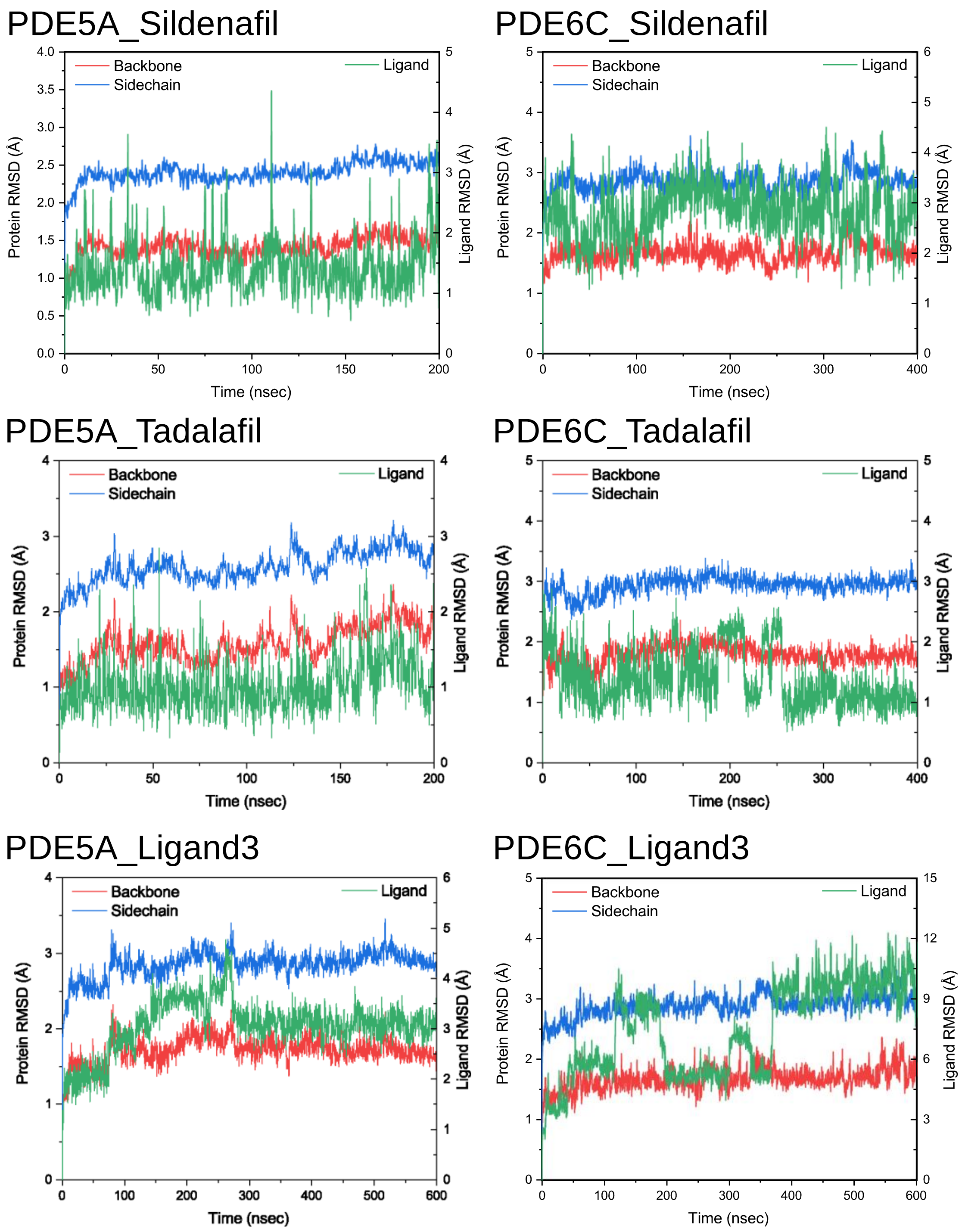

Figure S2 RMSD values of PDE5A and PDE6C complexes throughout the entire MD simulations

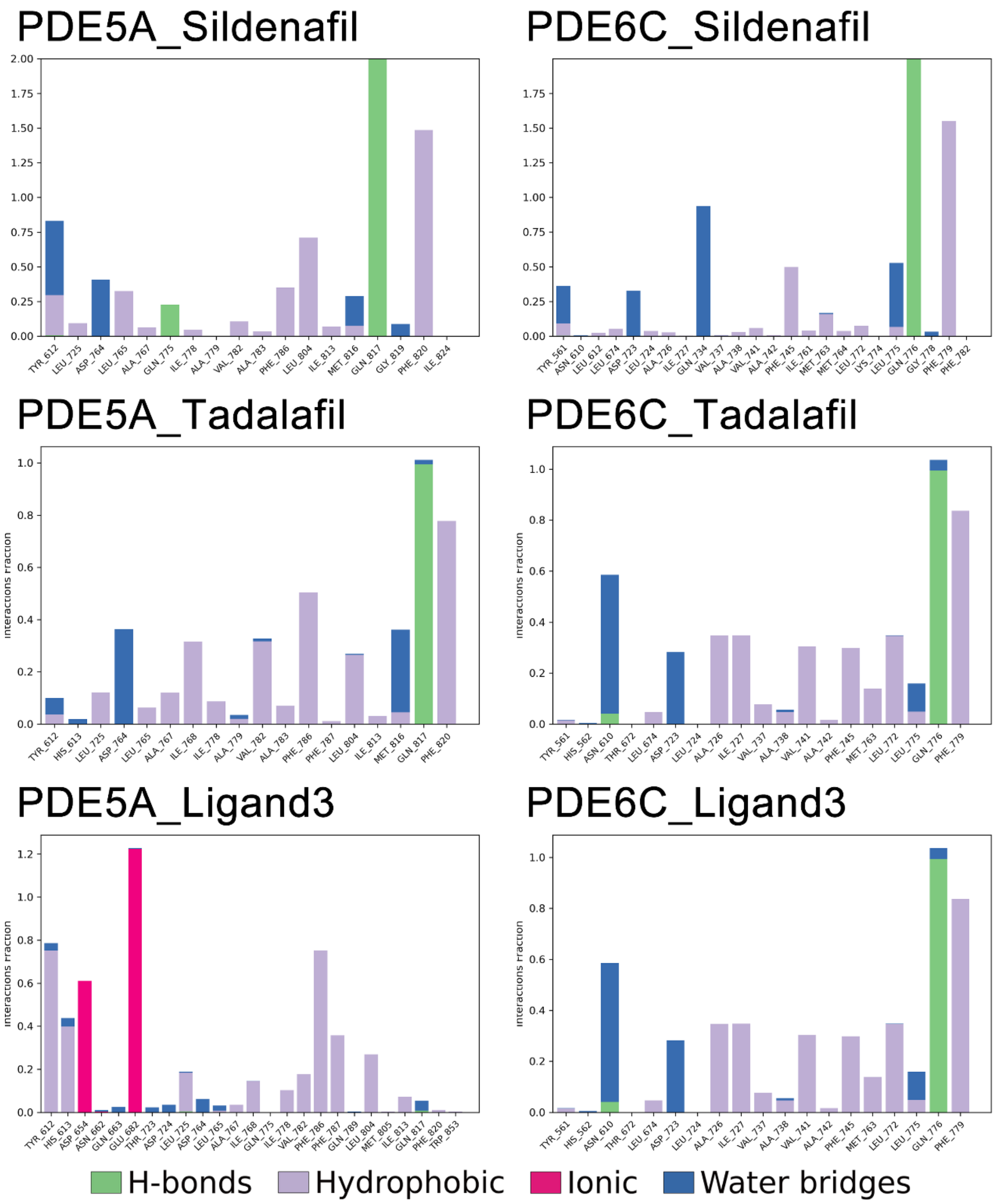

Figure S3 Contacts Histogram values of PDE5A and PDE6C complexes throughout the MD simulations

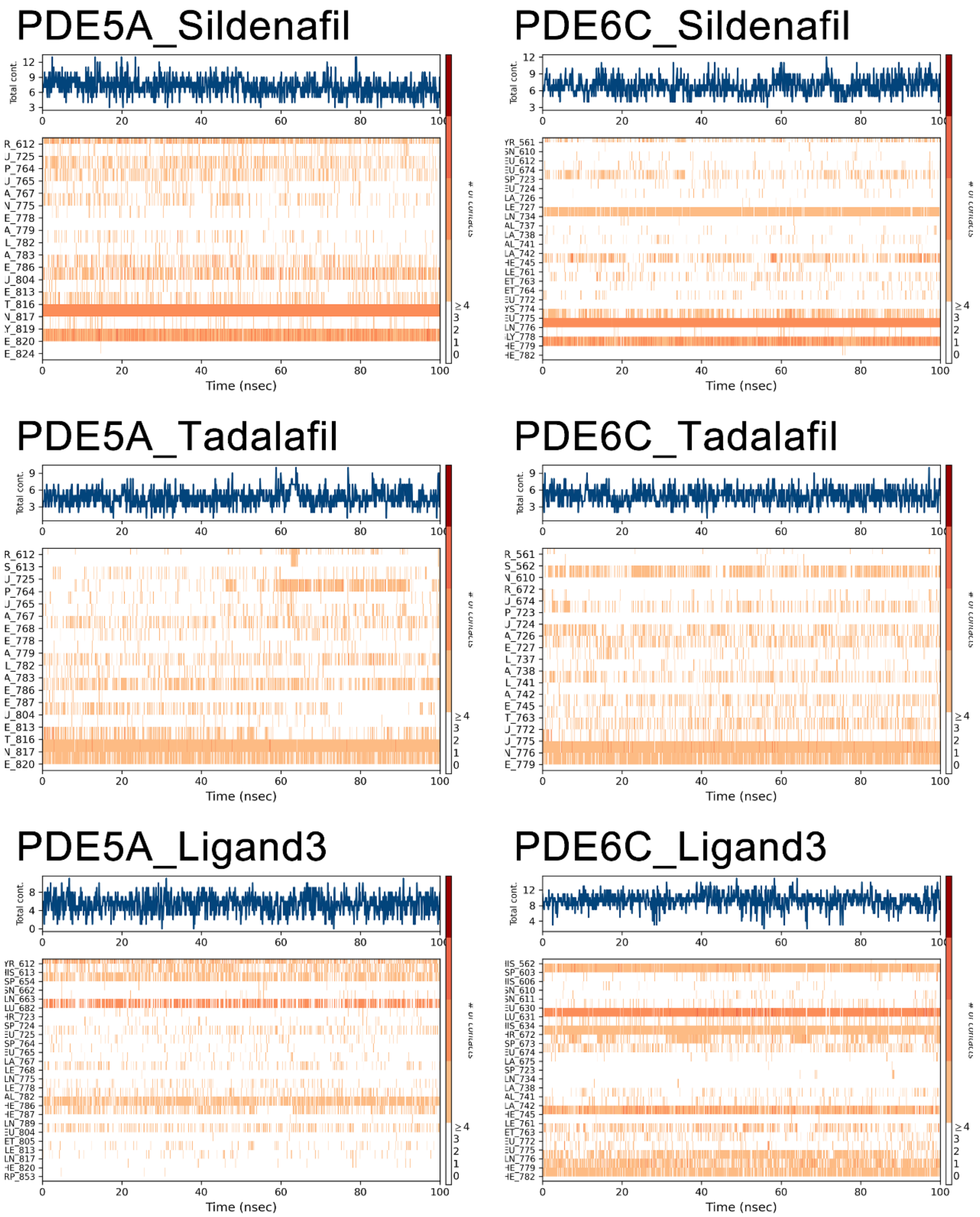

Figure S4 Contacts Timeline values of PDE5A and PDE6C complexes throughout the MD simulations
